# Unusual Presentation of Propionic Acidemia Mimicking Botulism in an Infant: A Case Report and Literature Review

**DOI:** 10.7759/cureus.66870

**Published:** 2024-08-14

**Authors:** Allison C Boland, Alexander Wind, Mohammad Alkhoujah

**Affiliations:** 1 Orthopedic Surgery, Wayne State University School of Medicine, Detroit, USA; 2 Pediatrics, Wayne State University School of Medicine, Detroit, USA; 3 Neurology, Henry Ford Health System, Detroit, USA

**Keywords:** mri, basal ganglia, pediatrics, neurology, infant, descending paralysis, botulinum, botulin toxin, propionic acidemia

## Abstract

Propionic acidemia (PA) is a rare metabolic disorder stemming from genetic mutations, often causing hyperammonemia, acidosis, and basal ganglia issues. Its symptoms range from vomiting to neurological abnormalities, with severe cases presenting in neonates. Neurological complications including stroke-like episodes are common, requiring immediate attention.

An eight-month-old boy with PA presented to the emergency department with respiratory distress, cough, and lethargy. Initial evaluation showed acidemia and elevated ammonia levels. He tested positive for rhinovirus and was diagnosed with acute viral bronchiolitis. While his respiratory symptoms improved, he developed neurological deficits, including hypotonia and weakness. Neurology consultations explored possible diagnoses such as botulism or acute inflammatory demyelinating polyneuropathy (AIDP). Imaging revealed basal ganglia abnormalities consistent with PA progression. Due to aspiration risk, he was transferred to the pediatric intensive care unit for supportive care. Despite unremarkable lumbar puncture and MRI results, new metabolic brain changes were noted, particularly in the basal ganglia. He was managed for weakness and feeding difficulties due to a metabolic stroke. After adjusting nutritional support and discussing long-term feeding options, he was discharged on day 29 with a nasogastric tube due to his inability to meet caloric goals orally.

Neurological complications in PA, such as basal ganglia abnormalities and stroke-like episodes, are well-documented. Our case illustrates how an acute respiratory illness can obscure underlying neurological deficits, leading to delayed diagnosis. Symptoms resembling other conditions, such as descending hypotonia in our case, broaden the differential diagnosis to include botulism toxicity and AIDP. This report demonstrates the variety of clinical features patients with PA can present with and the importance of working up a metabolic crisis in addition to conditions with overlapping symptoms.

## Introduction

Propionic acidemia (PA) is a rare autosomal recessive metabolic disorder resulting from mutations in the propionyl CoA carboxylase gene (*PCC*), impacting various systems within the human body [1]. It can frequently lead to episodes of hyperammonemia, metabolic acidosis, and infarction within the basal ganglia [2-4]. Propionyl-CoA is produced from the breakdown of several amino acids, including isoleucine, valine, threonine, methionine, odd-chain fatty acids, thymidine, uracil, and cholesterol, as well as potentially by gut bacteria. Propionyl-CoA is converted into methylmalonyl-CoA by the enzyme propionyl-CoA carboxylase (PCC) [5]. Methylmalonyl-CoA is then further converted into succinyl-CoA by methylmalonyl-CoA mutase. A deficiency or mutation in *PCC* leads to PA, while a deficiency or mutation in methylmalonyl-CoA mutase results in methylmalonyl aciduria [5].

The onset of this metabolic disorder spans from neonatal to late-onset, encompassing a spectrum of disease severity [6], and those with earlier onset had poorer prognosis [7]. PA affects 1:100,000-150,000 individuals [8].

In the classical neonatal-onset form, signs usually appear within the initial days of life, presenting as vomiting, dehydration, weight loss, temperature fluctuations, and neurological symptoms such as increased muscle tone, irritability, lethargy leading to coma, and seizures [8]. Laboratory assessments typically reveal pronounced and persistent metabolic acidosis and ketosis, increased anion gap, and elevated levels of ammonia [8]. Neurological complications [9] in PA can include stroke-like episodes, seizures, basal ganglia abnormalities manifesting in ataxia or disordered movement, extrapyramidal symptoms, brain atrophy, cardiomyopathy [10], and gastrointestinal difficulties. The classical neonatal onset of PA presents with distinct clinical features and laboratory findings indicative of a metabolic crisis, highlighting the need for prompt recognition and intervention. This report highlights an atypical presentation of PA in an infant which mimicked botulism or acute inflammatory demyelinating polyneuropathy (AIDP) due to his descending weakness and areflexia.

## Case presentation

An eight-month-old boy with a past medical history significant for PA presented to the emergency department with respiratory distress, cough, and lethargy. On arrival, the patient was afebrile, tachycardic at 140s-160s beats per minute, tachypneic at 40-70 respirations per minute, and well-saturated on room air at 96-100% oxygen saturation. Labs were significant for acidemia, -11 base excess, elevated ammonia, and anion gap (Tables 1, 2). Initial interventions included a high-flow nasal cannula and 1.5× maintenance IV fluids in addition to home medications carnitine and carglumic acid. The patient tested positive for rhinovirus and was diagnosed with acute viral bronchiolitis.

**Table 1:** Capillary blood gas laboratory values on initial presentation to the emergency department.

Lab parameter	Value	Reference range
pH	7.27	7.3–7.4
CO_2_	32	40–50 mmHg
HCO_3_	14.6	22–26 mEq/L
Base excess	-11	-4 to +2
Lactate	2.6	1.13–1.27

**Table 2:** Serum chemistry laboratory values on initial presentation to the emergency department.

Lab parameter	Value	Reference range
Lactic acid	1.2	0.4–2
Ammonia	88	16–53
Anion gap	22	5–15

Nutrition and genetic services were both consulted and actively provided recommendations for this case. On day two, the patient was weaned to room air which was tolerated well with no significant work of breathing. IV fluids and a special PA diet were continued to prevent further metabolic decompensation. On day three, the bronchiolitis symptoms resolved, but it was noted that the patient now had decreased truncal and head support, which were previously intact upon admission. Neurology was consulted on day four, and a full neurological examination revealed axial and peripheral hypotonia, drooling, head lag, proximal muscle weakness in the upper extremities, and absent deep tendon reflexes in the upper extremities and ankles bilaterally. At this time, the top differential diagnoses included botulism toxicity and AIDP, so serum botulinum toxin, GQ1B, GM1, GM2, and lumbar puncture were ordered. The medical laboratory tests GQ1B, GM1, and GM2 refer to specific antibodies or antigens associated with certain neurological conditions such as Miller-Fisher syndrome [11], Guillain-Barré syndrome [12], Tay Sachs [13], and Sandhoff disease [14].

**Figure 1:**
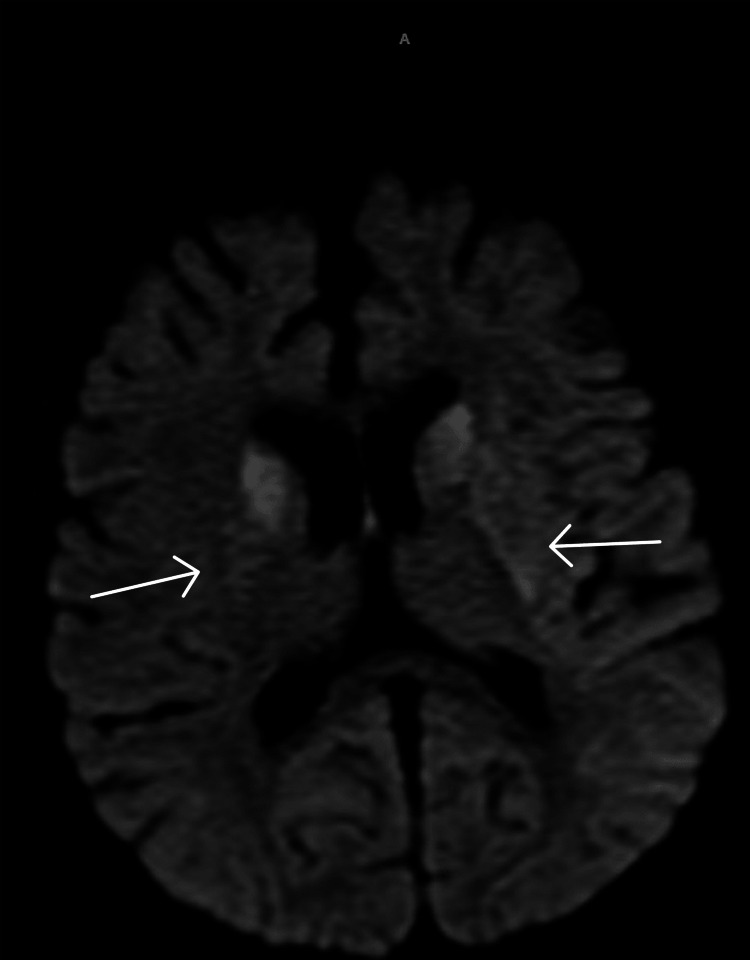
Diffusion-weighted image MRI showing bilateral symmetric areas of diffusion restriction mainly involving the basal ganglia including the caudate nucleus putamen and globus pallidus pallidum.

**Figure 2:**
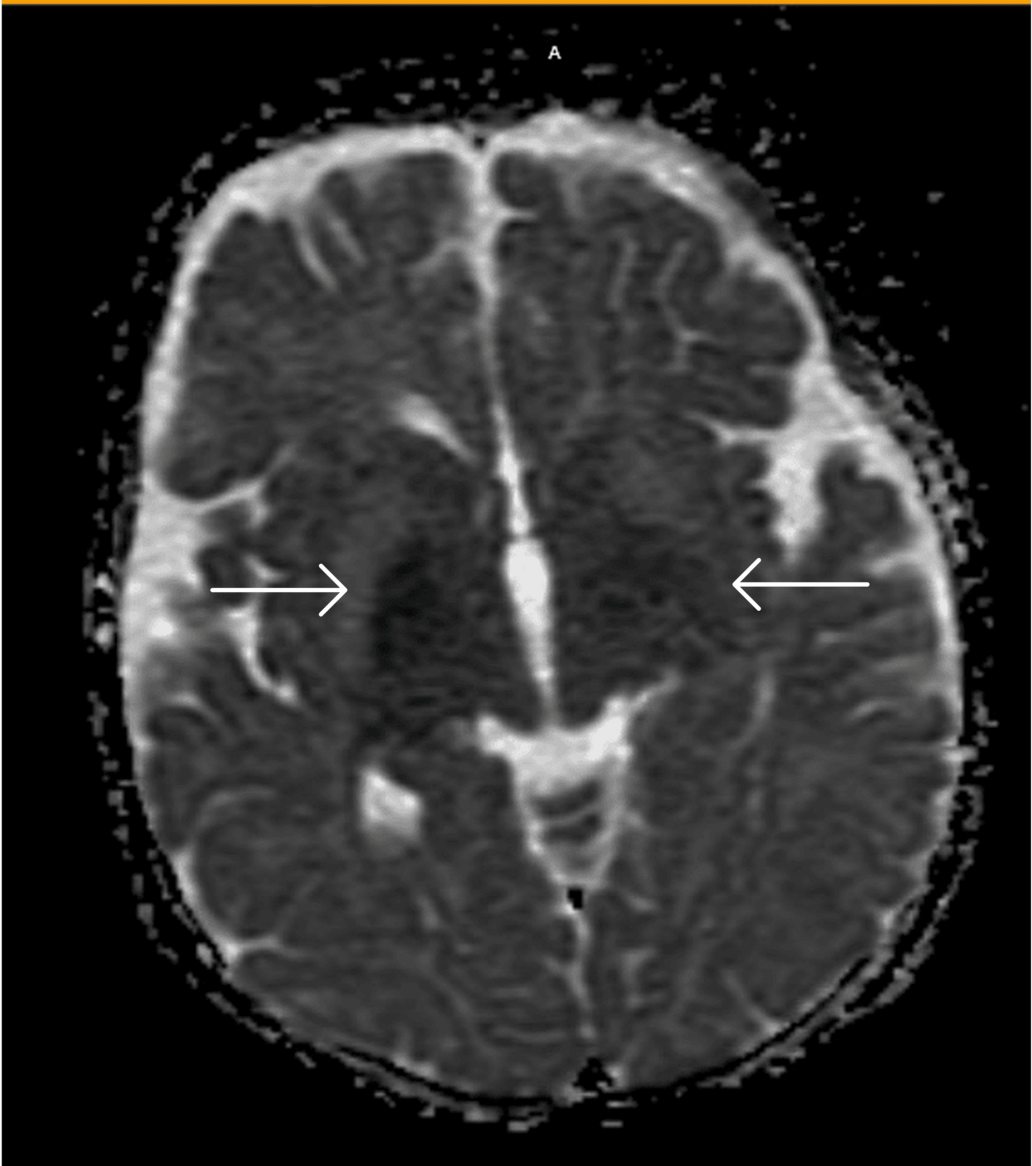
Apparent diffusion coefficient MRI showing bilateral symmetric areas of diffusion restriction mainly involving the basal ganglia including the caudate nucleus putamen and globus pallidus pallidum.

**Figure 3:**
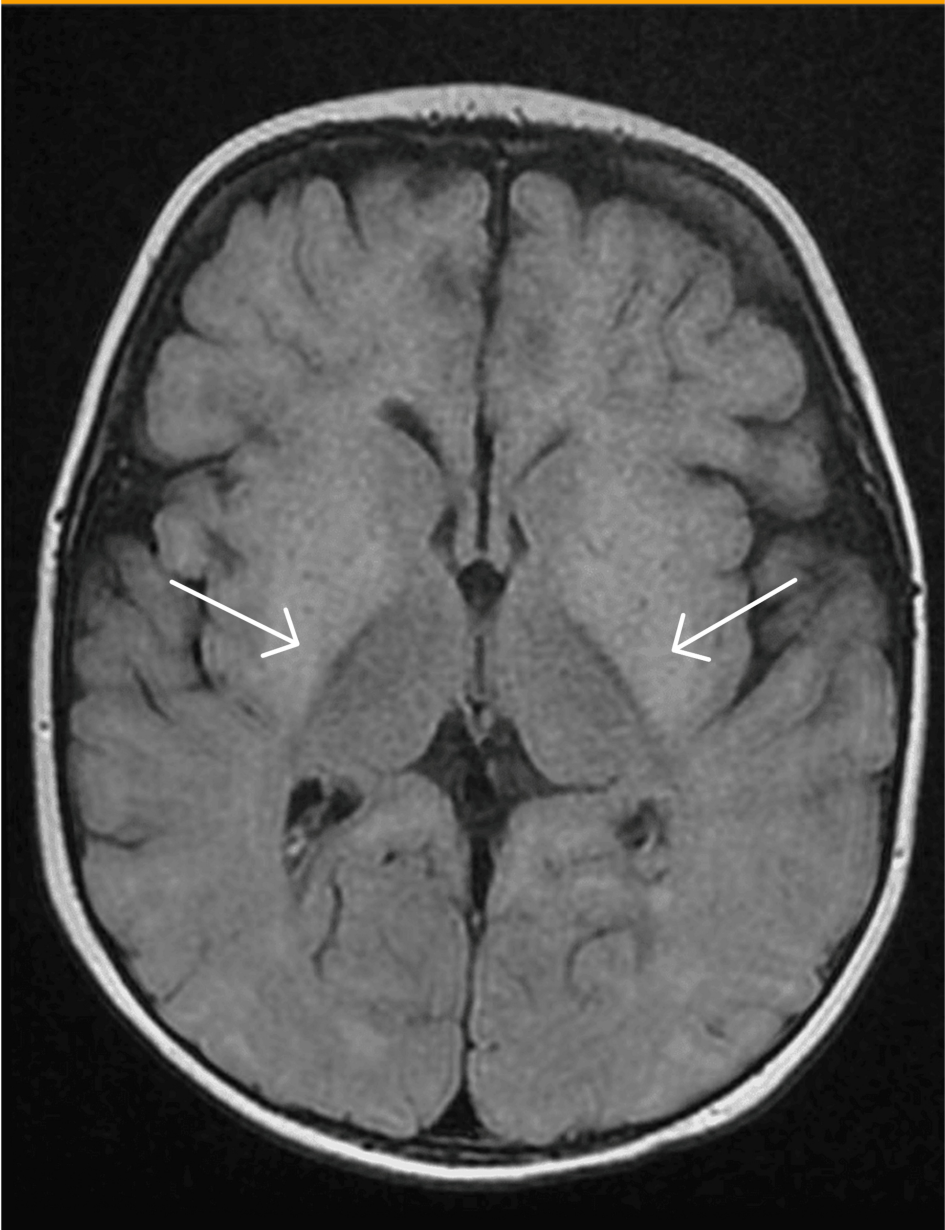
Fluid-attenuated inversion recovery MRI showing bilateral symmetric areas of diffusion restriction mainly involving the basal ganglia including the caudate nucleus putamen and globus pallidus pallidum.

On day five, the patient was transferred from the floor to the pediatric intensive care unit (PICU) due to the risk of aspiration secondary to increased drooling and pooled oral secretions. CT of the head was unremarkable, and a nasogastric (NG) tube was placed. The lumbar puncture results were unremarkable (Table 3) and subsequent MRI of the brain (day eight) showed diffusion restriction in basal ganglia bilaterally as well as volume loss in the subcortical space likely related to the progression of his metabolic illness (Figures 1-3). The patient remained clinically stable and tolerated NG tube feeds well and was transferred back from the PICU to the floor on day 10. At this time, the patient continued to have truncal weakness and head lag but was able to move all extremities and had no respiratory distress. The patient was admitted to the Physical Medicine and Rehabilitation service for the management of weakness and feeding difficulties secondary to metabolic stroke. From day 10 through day 24, the patient continued to tolerate NG tube feeds, although nutrition and IV fluid formulation were regularly modified based on daily labs, including urine ketones that fluctuated between 0 and 3+. The NG tube was pulled at the family’s request to attempt a PO (per oral) trial, but the patient was only able to intake a fraction of his caloric goal. Therefore, the team had discussions with the family about being discharged with the NG tube versus gastric tube insertion to meet nutrition goals and prevent further metabolic crises.

**Table 3:** Cerebrospinal fluid laboratory values.

Lab parameter	Value	Reference range
Lactic acid	2.4	1.1–2.8
Glucose	52	40–70
Protein	17	15–40
Cell count	0	<5

## Discussion

**Figure 4:**
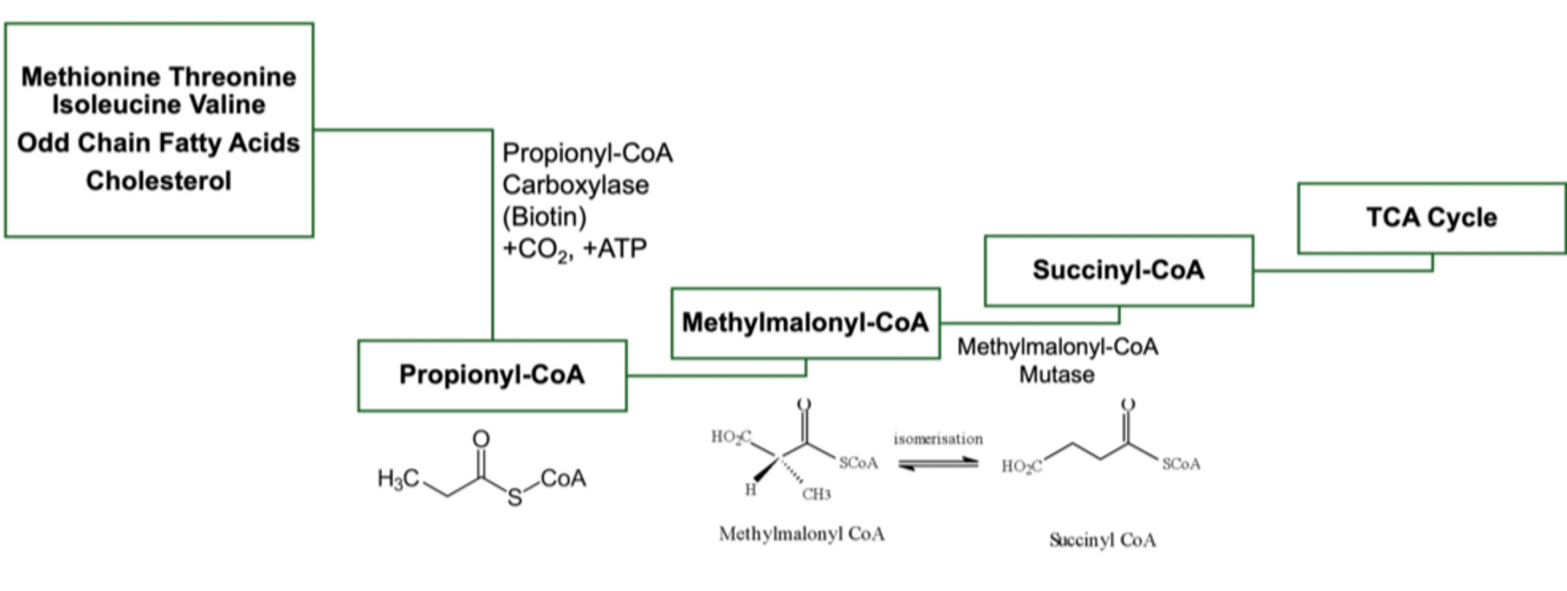
Propionyl-CoA metabolism.

Figure 4 illustrates the metabolism of propionyl-CoA.

Neuroimaging

Previous literature has investigated the neurological manifestations of PA demonstrating that PA manifestations can be seen on neuroimaging. In a 1999 retrospective analysis [15] of five patients with PA, follow-up MRI scans showed progressive basal ganglia degeneration, including atrophy and signal abnormalities in the caudate nuclei and putamina. Positron emission tomography scans initially displayed increased uptake in the basal ganglia and thalami, followed by decreased uptake in the basal ganglia as the disease progressed. In a 2018 retrospective analysis [16] of 14 patients in Saudi Arabia, it was discovered that the brain MRI scans of 5 out of 14 patients exhibited signal alterations in the basal ganglia. Additionally, Bergman et al. [17] studied three patients with PA; brain MRI revealed delayed myelination and cerebral atrophy in all cases, with bilateral signal abnormalities in the putamen and caudate nuclei, particularly observed in the patient diagnosed at nine months after displaying hypotonia and choreoathetoid movements following an upper respiratory tract infection. This presentation was similar to our eight-month-old patient who presented with acute-onset axial and peripheral hypotonia, excessive drooling, delayed head control, weakness in proximal muscles of the upper limbs, and bilaterally absent deep tendon reflexes after a respiratory infection. Neurological complications in PA, including basal ganglia abnormalities and stroke-like episodes, underscore the multisystem impact of the disorder and the complexity of its management. Extensive research on the neurological manifestations of PA highlights the detectability of PA-related abnormalities through neuroimaging techniques such as MRI, shedding light on the progressive nature of basal ganglia degeneration and providing insights into disease progression.

Stroke-like episodes in propionic acidemia patients

Another study by Almuqbil et al. [18] presented the case history of a patient with PA who experienced five episodes of acute hemiparesis between the ages of 3 and 11. Neuroimaging revealed basal ganglia signal changes years after the initial episode, and two episodes were accompanied by seizures. Despite occurring without apparent metabolic decompensation and lacking acute brain MRI findings, these hemiparetic events were consistent with metabolic strokes. If initial laboratory tests are normal or unclear, the study suggested comparing cerebrospinal fluid biochemistry with plasma levels to help identify signs of metabolic decompensation [18]. Additionally, urine organic acids and plasma acylcarnitines can also be useful. Magnetic resonance spectroscopy can further assist in detecting changes in metabolites. Hamilton et al. [19] described a nine-year-old with PA under good metabolic control at the time of death, who exhibited acute hemorrhagic lesions in the basal ganglia and thalamus, along with endothelial changes suggestive of blood-brain barrier breakdown. This patient upon autopsy was shown to have severe scarring of the pancreas and kidney failure, and the study suggested that her death was possibly linked to precending pancreatitis, although remaining uncertain. The etiology of the neurologic manifestations of PA is not well understood and can present differently. Cytotoxic damage to the central nervous system (CNS) can present like a stroke, similar to that of a four-year-old boy [20] with PA and an 11-year-old girl [21].

Comparison of propionic acidemia with other metabolic disorders

PA patients had more frequent basal ganglia lesions compared to patients with methylmalonic aciduria (Figure 4) or isovaleric aciduria [22], and numerous studies have indicated bilateral basal ganglia involvement [17,19,23].

Therapeutic interventions

Interestingly, Broomfield et al. [3] demonstrated the spontaneous and rapid resolution of acute basal ganglia changes in an untreated infant diagnosed with PA. Other studies have suggested liver transplants [2,6,10,24-26] and dietary management [27] as possible solutions. Despite the severity of PA-related neurological complications, spontaneous resolution of acute basal ganglia changes suggests potential for therapeutic interventions to mitigate disease progression. Early diagnosis and initiation of treatment for PA improve patient outcomes [10].

Clinical considerations

Our patient exhibited no ataxia, chorea, or movement disorder which is often seen in patients with PA [7]. Raimann et al. [28] reported the case of a six-day-old boy with PA who also presented with hypotonia but was noted to have hyperreflexia, unlike our patient. Our patient’s unique clinical presentation prompted discussion among the medical team, emphasizing the importance of considering atypical signs and symptoms even when a diagnosis of PA is known. While pursuing the workup for this patient, it became evident that earlier MRI evaluation of the CNS could have provided valuable insights. Performing an MRI earlier might have streamlined the diagnostic process, limiting the need for extensive workup. However, the clinical circumstances dictated the course of action, emphasizing the urgency of investigating lower motor neuron involvement promptly, particularly considering conditions such as Guillain-Barré syndrome, which can progress rapidly and lead to respiratory distress. His presentation with lower motor neuron signs after an upper respiratory infection required a broader differential diagnosis. Our patient’s diminished reflexes were recognized as more likely a metabolic manifestation rather than a primary neurological deficit.

## Conclusions

This case presentation illustrates the challenges in diagnosing and managing PA-related neurological complications in the setting of upper respiratory infection. Here, we show how an infant with PA can present with a similar disease course to botulism with areflexia. Documenting this case provides valuable clinical insights for healthcare professionals, alerting them to the potential for PA to present with unusual symptoms such as those resembling botulinum toxin poisoning or AIDP. This knowledge can help clinicians make more informed diagnostic and treatment decisions in similar cases in the future.

## References

[1] Cao LX, Hu WZ, Dong W (2023). Neuropathological report of propionic acidemia. Neuropathology.

[2] Barshes NR, Vanatta JM, Patel AJ, Carter BA, O'Mahony CA, Karpen SJ, Goss JA (2006). Evaluation and management of patients with propionic acidemia undergoing liver transplantation: a comprehensive review. Pediatr Transplant.

[3] Broomfield A, Gunny R, Prabhakar P, Grunewald S (2010). Spontaneous rapid resolution of acute basal ganglia changes in an untreated infant with propionic acidemia: a clue to pathogenesis?. Neuropediatrics.

[4] Shuaib T, Al-Hashmi N, Ghaziuddin M (2012). Propionic acidemia associated with visual hallucinations. J Child Neurol.

[5] Bodamer OA (2024). Organic acidemias: an overview and specific defects. UpToDate.

[6] Shchelochkov OA, Carrillo N, Venditti C (1993). Propionic acidemia. GeneReviews(®).

[7] Surtees RA, Matthews EE, Leonard JV (1992). Neurologic outcome of propionic acidemia. Pediatr Neurol.

[8] Baumgartner MR, Hörster F, Dionisi-Vici C (2014). Proposed guidelines for the diagnosis and management of methylmalonic and propionic acidemia. Orphanet J Rare Dis.

[9] Pena L, Franks J, Chapman KA (2012). Natural history of propionic acidemia. Mol Genet Metab.

[10] Ehrenberg S, Walsh Vockley C, Heiman P, Ammous Z, Wenger O, Vockley J, Ghaloul-Gonzalez L (2022). Natural history of propionic acidemia in the Amish population. Mol Genet Metab Rep.

[11] Lo YL (2007). Clinical and immunological spectrum of the Miller Fisher syndrome. Muscle Nerve.

[12] Willison HJ, O'Hanlon G, Paterson G (1997). Mechanisms of action of anti-GM1 and anti-GQ1b ganglioside antibodies in Guillain-Barré syndrome. J Infect Dis.

[13] Tsuji D, Higashine Y, Matsuoka K, Sakuraba H, Itoh K (2007). Therapeutic evaluation of GM2 gangliosidoses by ELISA using anti-GM2 ganglioside antibodies. Clin Chim Acta.

[14] Myerowitz R, Lawson D, Mizukami H, Mi Y, Tifft CJ, Proia RL (2002). Molecular pathophysiology in Tay-Sachs and Sandhoff diseases as revealed by gene expression profiling. Hum Mol Genet.

[15] Al-Essa M, Bakheet S, Patay Z (1999). 18Fluoro-2-deoxyglucose (18FDG) PET scan of the brain in propionic acidemia: clinical and MRI correlations. Brain Dev.

[16] AlGhamdi A, Alrifai MT, Al Hammad AI, Al Mutairi F, Alswaid A, Eyaid W, Alfadhel M (2018). Epilepsy in propionic acidemia: case series of 14 Saudi patients. J Child Neurol.

[17] Bergman AJ, Van der Knaap MS, Smeitink JA, Duran M, Dorland L, Valk J, Poll-The BT (1996). Magnetic resonance imaging and spectroscopy of the brain in propionic acidemia: clinical and biochemical considerations. Pediatr Res.

[18] Almuqbil M, Chinsky JM, Srivastava S (2019). Metabolic strokes in propionic acidemia: transient hemiplegic events without encephalopathy. Child Neurol Open.

[19] Hamilton RL, Haas RH, Nyhan WL, Powell HC, Grafe MR (1995). Neuropathology of propionic acidemia: a report of two patients with basal ganglia lesions. J Child Neurol.

[20] Karall D, Haberlandt E, Schimmel M (2011). Cytotoxic not vasogenic edema is the cause for stroke-like episodes in propionic acidemia. Neuropediatrics.

[21] Scholl-Bürgi S, Haberlandt E, Gotwald T (2009). Stroke-like episodes in propionic acidemia caused by central focal metabolic decompensation. Neuropediatrics.

[22] Nizon M, Ottolenghi C, Valayannopoulos V (2013). Long-term neurological outcome of a cohort of 80 patients with classical organic acidurias. Orphanet J Rare Dis.

[23] Johnson JA, Le KL, Palacios E (2009). Propionic acidemia: case report and review of neurologic sequelae. Pediatr Neurol.

[24] Davison JE, Davies NP, Wilson M (2011). MR spectroscopy-based brain metabolite profiling in propionic acidaemia: metabolic changes in the basal ganglia during acute decompensation and effect of liver transplantation. Orphanet J Rare Dis.

[25] Fraser JL, Venditti CP (2016). Methylmalonic and propionic acidemias: clinical management update. Curr Opin Pediatr.

[26] Zhang Y, Peng C, Wang L (2023). Prevalence of propionic acidemia in China. Orphanet J Rare Dis.

[27] Sutton VR, Chapman KA, Gropman AL (2012). Chronic management and health supervision of individuals with propionic acidemia. Mol Genet Metab.

[28] Raimann E, Cornejo V, Perales CG, Colombo M (1990). Propionic acidaemia: two cases in Chile. J Inherit Metab Dis.

